# Protonation- and substrate-regulated dimer opening couples brain-type creatine kinase to vesicular and actin-remodeling membranes

**DOI:** 10.21203/rs.3.rs-10582165/v1

**Published:** 2026-09-30

**Authors:** Samantha Gies, Nolan K. McLaughlin, Ahmed Shubbar, Chen Kong, Tianqi Wu, Sana Khan, Alessandro Ustione, Ian Miller, David W. Piston, Mahmoud Moradi, Michael L. Gross, Reza Dastvan

**Affiliations:** 1 Department of Biochemistry and Molecular Biology, Saint Louis University School of Medicine, St. Louis, MO, USA; 2 Department of Chemistry, Washington University in St. Louis, St. Louis, MO, USA; 3 Department of Chemistry and Biochemistry, University of Arkansas, Fayetteville, AR, USA; 4 Department of Cell Biology and Physiology, Washington University School of Medicine, St. Louis, MO, USA; 5 Center for Applied AI for Protein Dynamics, Vanderbilt University, Nashville, TN, USA; 6 These authors contributed equally to this work.

## Abstract

Brain-type creatine kinase (CK-BB) buffers local ATP demand through reversible phosphotransfer between ATP and phosphocreatine, yet how this soluble metabolic enzyme couples to dynamic membrane compartments remains unclear. Here, we integrate immunofluorescence microscopy, DEER spectroscopy, hydrogen-deuterium exchange and native mass spectrometry, DEER- and AlphaFold-guided modeling, and long-timescale molecular dynamics to define the acid- and substrate-regulated conformational landscape of human CK-BB. Acidification redistributes endogenous and recombinant CK-BB from a diffuse cytosolic pool to punctate vesicular structures and membrane ruffles. DEER and modeling reveal a spatially asymmetric dimer in which the convex surface remains comparatively restrained, whereas the concave catalytic-regulatory surface samples pH- and substrate-dependent conformational intermediates. We identify progressive dimer opening as a novel regulatory mechanism by which acidic pH and substrate binding increase dynamics across the convex surface and NTD–NTD interface and sensitize the His191/Ser199 module to graded pH-dependent exchange. Substrate binding buffers acid-induced deprotection while preserving localized dynamics near this regulatory interface. Together, these data support a model in which protonation and substrate occupancy generate membrane-competent CK-BB conformations, coupling local ATP regeneration to curved vesicular and actin-remodeling membranes.

## Introduction

Creatine kinases (CKs) form a phosphagen circuit that buffers ATP and ADP in tissues and subcellular compartments with high and fluctuating energy demand^1–6^. Cytosolic brain-type creatine kinase (CK-BB) functions as a homodimer that catalyzes reversible phosphoryl transfer between ATP and creatine (Cr), producing ADP, H^+^ and phosphocreatine (PCr). This reaction supports rapid ATP regeneration at sites of localized energy consumption, including those of neuronal processes, ion-transport microdomains, actomyosin assemblies, cortical membrane compartments, and other CK-BB-enriched subcellular microdomains^1,4–10^.

CK-BB has emerged as a regulator of cell morphology, motility and metastatic disease. Its cellular distribution is altered in metastatic cancer cells^3,11–13^, and CK-BB has been detected extracellularly or as a serum-associated marker in several cancers, including colorectal, ovarian, neuroblastoma, prostatic and lung cancer^11,12,14,15^. CK-BB-dependent phosphagen metabolism has also been linked to metastatic survival, hypoxia, invasion and therapeutic vulnerability^11,13,16–23^. Yet, despite its extracellular detection and functional association with metastatic energetics^11,19^, CK-BB lacks a canonical secretion signal peptide. These observations place CK-BB within the broader context of unconventional protein secretion (UPS), in which leaderless cytosolic proteins bypass the endoplasmic reticulum and Golgi apparatus through pathways that often involve lipid binding, membrane insertion, or trafficking through endosomal and secretory intermediates, particularly under cellular stress^24,25^. Consistent with this framework, CK-BB transiently accumulates in actin-rich membrane ruffles and phagocytic cups, and loss or inhibition of CK-BB activity impairs cell spreading, migration and complement-mediated phagocytosis^9,10,26^. Together, these findings suggest that CK-BB function depends not only on catalytic chemistry, but also on regulated localization, stress-dependent membrane access, and conformational adaptability.

Prior biochemical observations further support the potential for CK-BB membrane engagement. CK isoforms interact with phospholipid monolayers^27^, and CK-BB or CK activity has been associated with synaptosomal membranes and synaptic vesicles^6,28,29^. In Alzheimer’s disease brain and oxidative-stress models, CK-BB modifications reduce activity, impair nucleotide binding, and coincide with aberrant membrane partitioning or focal creatine deposits^30–32^. Together, these findings position CK-BB as a soluble enzyme whose disease-associated functions may depend not only on catalytic regulation, but also on controlled access to membrane-proximal compartments.

Acidification is particularly relevant to CK-BB biology. Hypoxic and metabolically stressed metastatic cancer cells experience altered intracellular and extracellular pH, and acidic microenvironments promote metastasis, invasion, secretion, vesicle trafficking and cytoskeletal remodeling in cancer and inflammatory contexts^33–36^. Disrupted intracellular pH homeostasis is also associated with neuroinflammatory and neurodegenerative states, including neuronal cytosolic acidification and excessive endosomal acidification in AD-relevant astrocytes^37,38^. In parallel, endosomal and secretory compartments maintain regulated pH gradients that can reshape protein–membrane interactions. Acute extracellular acidification, therefore, provides a tractable approach to test whether an acid-responsive CK-BB state is sufficient to redistribute the enzyme from a diffuse cytosolic pool to vesicular or cortical membrane compartments. For a soluble enzyme enriched at membrane ruffles and vesicular structures, a central question is whether acidification directly remodels the CK-BB dimer to expose or stabilize membrane-engaging conformations, and whether substrate binding reshapes this response.

High-resolution structures of human and chicken CK-BB have captured the dimeric ligand-free protein^39,40^ and ligand-bound states^41^, showing that nucleotide and transition-state analogue binding promotes active-site closure and rearranges catalytic loops required for substrate recognition and phosphoryl transfer^41^. Complementary structural and biochemical studies have identified catalytically important flexible loops, active-site histidines, nucleotide-binding arginine residues, and loop movements that occlude the active site during catalysis^42–45^. However, the structural and dynamic basis by which CK-BB adapts to acidification, substrate binding, and membrane-proximal environments remains poorly understood. This gap is important because CK-BB operates in settings where pH, substrate availability, ionic composition, and membrane curvature can vary locally, including hypoxic tumors, secretory and vesicular compartments, synaptic membranes, and actin-rich cortical domains.

Here, we use an integrated structural-dynamics platform to define how acidic pH and ligand binding remodel CK-BB. Immunofluorescence shows that acidic conditions redistribute CK-BB to vesicular and membrane-ruffle compartments^26,46^. Double electron-electron resonance spectroscopy (DEER, also known as PELDOR)^47–52^ maps inter-protomer conformational equilibria across the convex and concave faces of the dimer, while DEER- and AlphaFold-guided modeling^53–56^ defines the CK-BB conformational landscape and reveals how protonation and ligand binding bias its energy landscape. Hydrogen-deuterium exchange mass spectrometry (HDX-MS)^57–62^ provides comprehensive peptide-level readouts of local backbone dynamics and solvent exposure, and molecular dynamics simulations^63–67^ connect these exchange signatures to structural motion. Together, these approaches reveal a pH- and ligand-gated CK-BB energy landscape in which dimer opening and selective remodeling of convex and concave surfaces couple local ATP regeneration to curved vesicular and actin-remodeling membranes.

## Results

### Acidification recruits CK-BB to vesicles and membrane ruffles

Because CK-B is recruited to actin-rich cortical microdomains, supports local ATP generation during actin remodeling, and is implicated in extracellular metabolic energetics, we first tested whether acidification changes the cellular distribution of CK-BB. HCT116 colorectal carcinoma and HEK293 cells expressing endogenous CK-BB or recombinant FLAG-tagged CK-BB were incubated at neutral pH and pH 5.5 and analyzed by immunofluorescence (Fig. 1). At pH 7.2, CK-BB was largely diffuse and cytosolic in both cell types, consistent with its canonical role as a soluble phosphotransfer enzyme (Fig. 1a). In contrast, a change to pH 5.5 increased punctate and vesicular staining and enriched CK-BB at membrane ruffles (Fig. 1b), a pattern observed for endogenous CK-BB and for recombinant FLAG-CK-BB detected with either anti-CK-BB or anti-FLAG antibodies.

These cellular data extend prior observations that CK-B accumulates in phagocytic cups, membrane ruffles, and other actin-rich protrusions^9,10,26^. They also align with the UPS-related premise that stress-associated acidification, vesicular trafficking, membrane curvature, and actin remodeling can provide routes for leaderless cytosolic proteins to access membrane-proximal compartments^24,25^. Although these imaging experiments do not by themselves demonstrate secretion, membrane insertion, or translocation, they show that acidification alone is sufficient to bias CK-BB toward punctate vesicular and cortical membrane compartments. This provided the cellular rationale for testing whether protonation and substrate binding remodel the CK-BB dimer in ways that could favor membrane-proximal engagement.

### Functional and structural integrity of CK-BB constructs

For DEER measurements, single-cysteine substitutions were introduced into a cysteine-less (CL) CK-BB background to enable site-specific spin labeling. The five native cysteines were replaced by residues found in homologs or by substitutions previously shown to preserve activity and stability, yielding CL CK-BB^68,69^. Although the conserved Cys283, likely through its deprotonated thiolate state, contributes to maximal creatine kinase activity, current evidence does not support a direct catalytic role or a role in substrate-induced conformational changes^70^. We assessed the functional integrity of the CL construct relative to wild-type (WT) CK-BB using a creatine kinase activity assay that quantifies CK-dependent ATP generation from phosphocreatine and ADP. Mutation of Cys283 to serine strongly reduced activity, whereas the C283D-containing CL background retained measurable activity and was selected for DEER analyses (Supplementary Fig. 1a). Importantly, restoring Cys283 in the CL background, as in the CL^D283C^ construct, showed that mutation of the remaining native cysteines did not impair catalytic activity. Consistent with the pH-dependent reversibility of the CK reaction, WT CK-BB activity in the ATP-generating direction was higher at mildly acidic pH than at pH 7.3 (Supplementary Fig. 1b), supporting the functional relevance of the pH range used for structural-dynamics measurements^70^. Far-UV circular dichroism spectra of WT, CL and spin-labeled mutants were closely similar, confirming that cysteine substitutions and spin labeling did not perturb global secondary structure (Supplementary Fig. 1c). The complementary HDX-MS experiments were performed with WT protein.

### The convex and concave faces of CK-BB sample distinct conformational equilibria

To define how pH and substrate binding reshape the CK-BB dimer, we placed inter-protomer spin-label reporters across two major surfaces: the convex face, which spans the NTD dimer interface and C-terminal β-sheet core, and the concave face, which contains active-site and regulatory elements (Figs. 2 and 3). Ligand-bound CK-BB was prepared with 10 mM MgATP, 0.8 mM ADP, 20 mM PCr and 10 mM Cr to approximate a substrate-rich phosphagen-buffering state and promote occupancy of CK-BB ligand-coupled conformations^71^. On the convex surface, most reporter pairs generally showed narrow distance distributions and limited redistribution between pH 7.3 and 6.5 in both apo and ligand-bound states (Fig. 2 and Supplementary Fig. 2). More pronounced changes were observed primarily at pH 5.5, indicating that the convex surface is comparatively restrained under neutral and mildly acidic conditions but becomes more responsive upon stronger acidification.

The concave face displayed a markedly different behavior. Reporters surrounding the Ser199-containing region and neighboring active-site elements sampled multiple conformational intermediates that redistributed with both pH and ligand binding (Fig. 3 and Supplementary Fig. 3). Several reporter pairs resolved three distance populations: two relatively narrow, well-defined components and a broader, more dynamic population that became more prominent under acidic conditions, particularly at pH 5.5. Although some experimental components overlapped with distances predicted from AlphaFold3, X-ray, and cryo-EM structures, the DEER data revealed broader ensembles and intermediate states that were not captured by any single model.

Together, these results show that CK-BB is dynamically polarized. The convex face remains comparatively constrained across most conditions, whereas the concave face, particularly the region containing the nucleotide-binding residue His191 and the putative Ser199 phosphorylation site, is strongly coupled to protonation and ligand occupancy. This face-specific redistribution of conformational equilibria provides a structural basis for how acidic pH and substrate binding may remodel CK-BB toward membrane-engaging states.

### Protonation and ligand binding reveal dimer opening as a regulatory mechanism

We next translated the DEER-derived distance constraints into structural ensembles using DEERFold^53^, a fine-tuned AlphaFold2-based network^55^ that incorporates DEER distance distributions as distogram inputs to predict spin-label-constrained conformations. Single-Gaussian approximations of the stabilized distance components observed under each condition were used as DEERFold restraints. Principal component analysis resolved two dominant collective motions of the CK-BB dimer (Fig. 4a). PC1 described lateral dimer expansion, whereas PC2 captured hinge-like opening and closure, as illustrated by the corresponding morphing trajectories (Supplementary Videos 1 and 2). Within this conformational space, ligand-bound CK-BB at pH 5.5 shifted most strongly toward the laterally expanded, open ensemble. Ligand-bound CK-BB at pH 7.3 occupied an intermediate region, whereas apo ensembles remained comparatively closed (Fig. 4a). Using an independent refinement strategy, we refined the unbiased AlphaFold3 (AF3) model^56^ with the same DEER distance constraints, yielding similar conformational models (Fig. 4b and Supplementary Figs. 4 and 5)^54^.

These DEER-guided conformational trends were independently supported by molecular dynamics simulations. Across triplicate 1-μs simulations, dimer-opening angle distributions shifted toward larger opening angles under acidic conditions and upon ligand binding, with the most open conformations enriched in the ligand-bound acidic state (Figs. 4c and 4d). The convergence of DEER-guided structural ensembles, AF3-refined models and MD-derived opening angles indicates that CK-BB does not transition between two rigid endpoints. Instead, the dimer samples a continuum of pH- and ligand-dependent open states, in which protonation and ligand binding progressively bias the conformational landscape toward dimer expansion and hinge-like opening.

### HDX-MS resolves region-specific acid-enhanced CK-BB dynamics

HDX-MS provides a complementary, label-free approach for mapping CK-BB dynamics because backbone amide exchange reports local hydrogen bonding, conformational dynamics, and solvent accessibility at peptide resolution^72–75^. Faster exchange generally reflects increased local flexibility and/or solvent exposure, whereas slower exchange indicates more protected, hydrogen-bonded or structurally restrained regions. Because intrinsic amide exchange is strongly pH-dependent, labeling times were adjusted across pH conditions before deuterium uptake differences were interpreted as conformational or accessibility changes^58,59^. This makes HDX-MS particularly powerful for connecting acid-dependent structural remodeling to membrane-binding-competent CK-BB conformations^57–61^.

Native MS confirmed that CK-BB remains predominantly dimeric under the conditions used for structural analysis. The dominant native species was observed at 85,851 ± 1 Da, consistent with stable CK-BB dimer formation, whereas the smaller monomeric signal likely reflects partial gas-phase dissociation during ionization (Supplementary Figs. 6a and 6b). A minor +64 Da dimeric species was also detected, consistent with an unidentified post-translational or chemical modification. Denaturing MS yielded a monomeric mass of 42,925 Da, in close agreement with the calculated CK-BB mass of 42,925.5 Da, with minor higher-mass species at +98, +198 and +315 Da (Supplementary Figs. 6c and 6d). Together with native MS showing that CK-BB remains predominantly dimeric, the HDX-MS data did not show the broad protection pattern expected for aggregation or precipitation, supporting the interpretation that acidification promotes CK-BB redistribution rather than nonspecific aggregation.

We used HDX-MS to localize regions of CK-BB that undergo pH-dependent changes in backbone exchange. Because intrinsic exchange slows at lower pH, labeling times were adjusted to compare equivalent intrinsic-exchange windows across conditions (Supplementary Table 1). Pepsin digestion yielded 170 identified peptides, 100% sequence coverage, an average peptide length of 13.4 residues and a redundancy of 6.4, supporting robust peptide-level analysis (Supplementary Fig. 7). Volcano plots and peptide coverage maps showed that decreasing pH progressively increased both the magnitude and statistical significance of deuterium uptake in apo CK-BB relative to pH 7.3 (Figs. 5a–d). This response was modest and localized at pH 6.5, broader at pH 6.0 and widespread at pH 5.5. Ligand-bound CK-BB also showed acid-dependent increases in exchange, although the affected regions differed modestly from those in the apo state (Figs. 5e and 5f).

These increases in deuterium uptake support condition-dependent changes in local backbone dynamics and/or solvent exposure. Acidification broadly enhanced CK-BB backbone exchange while preserving strong regional specificity. The top of the convex dimer surface showed pronounced pH-dependent increases in exchange, whereas peptides along the lateral convex surfaces remained comparatively protected (Fig. 6). In apo CK-BB, exchange across the top convex surface changed modestly at pH 6.5 but increased markedly at pH 5.5, consistent with a threshold-like acid-induced transition. Ligand binding sensitized this region to pH, producing modest but graded increases in exchange across pH 7.3, 6.5 and 5.5. This HDX-MS pattern closely parallels the DEER measurements, which show that convex-surface reporters remain comparatively restrained with limited pH- or ligand-dependent redistribution except under stronger acidification (Fig. 2).

### Ligand binding sensitizes the His191/Ser199 concave-surface module to pH

HDX-MS identified a second major pH-responsive region on the concave surface of CK-BB, centered on the nucleotide ribose-interacting residue His191 and Ser199, a consensus ERK1/2 mitogen-activated protein kinase (MAPK) and putative AMP-activated protein kinase (AMPK) phosphorylation site (Fig. 7)^71,76^. His191 lies within the nucleotide-binding environment, whereas Ser199 is positioned adjacent to a short helix connected to the C-terminal β-sheet core (Fig. 7a). In apo CK-BB, peptides spanning this region showed only modest exchange differences between pH 7.3 and 6.5 but markedly increased deuterium uptake at pH 5.5, consistent with a threshold-like acid-induced increase in local backbone dynamics and/or solvent exposure.

Ligand binding reshapes this response. Rather than uniformly protecting the His191/Ser199 region, substrate binding sensitized it to pH, producing graded increases in exchange across pH 7.3, 6.5 and 5.5 (Fig. 7c). The strongest responses were observed in peptides spanning residues 174–193 and 181–193, whereas shorter peptides centered on residues 188–193 showed more limited changes, suggesting local protection within the His191-containing segment (Supplementary Fig. 11). Flanking peptides, including residues 158–173 and 203–226, also displayed acid-enhanced exchange (Fig. 7d and Supplementary Fig. 11), indicating that pH-dependent backbone dynamics extend beyond the central His191/Ser199 site across the surrounding concave surface.

Together with the DEER data (Fig. 3), which show pH- and ligand-dependent redistribution of conformational intermediates across this concave face, these HDX-MS results identify the His191/Ser199 region as a ligand-sensitized regulatory module in which protonation and substrate occupancy are coupled to inter-protomer ensemble remodeling.

### Molecular dynamics connect acid-sensitive HDX regions to dynamic structural elements

To determine whether HDX-sensitive regions correspond to intrinsically flexible segments or to structural elements that become dynamically remodeled in response to acidification and ligand binding, we performed triplicate 1-μs molecular dynamics simulations of apo and ligand-bound CK-BB at pH 7.3 and pH 5.5. Across all four states, Cα root-mean-square deviation (RMSD) traces showed overall structural stability, with the greatest conformational variability observed in the ligand-bound acidic state (Fig. 8d). Per-residue root-mean-square fluctuation (RMSF) profiles further showed that many acid- and ligand-sensitive HDX regions either coincide with locally flexible segments or lie adjacent to dynamic elements that could modulate solvent exposure (Fig. 8e). For example, residues 335–348, which lie within the largely buried CTD β-sheet core, are adjacent to a region with increased dynamics under acidic conditions (circled, Fig. 8e), potentially enhancing their solvent accessibility. The NTD–NTD dimer interface becomes more dynamic in the ligand-bound acidic state than in the apo state, potentially promoting sampling of more open ligand-bound conformations.

Three regions are particularly informative. First, the conserved flexible loop near the NTD interface, containing His66, Ile69 and Val72, showed both increased deuterium uptake under acidic conditions and local mobility in MD simulations (Figs. 8a and 8e and Supplementary Figs. 8–10). Peptides spanning residues 52–73 and 72–90 displayed a threshold-like acid-induced increase in exchange in apo CK-BB, whereas ligand-bound CK-BB showed more graded pH-dependent exchange across several overlapping peptides. This ligand-sensitized behavior extended to additional functional regions highlighted in Supplementary Fig. 8, including peptide regions containing Arg130, Arg132, Glu232, Arg236, Cys283, Arg292 and His296, indicating that substrate binding broadly tunes the pH responsiveness of catalytic and substrate-binding elements.

Second, the concave catalytic surface, including the His191/Ser199 region and neighboring Glu232, Cys283, Arg292 and His296 sites, contain several pH-responsive segments (Figs. 7 and 8b and Supplementary Figs. 11–13). Peptides spanning residues 207–226 and 251–281 showed acid-enhanced exchange, whereas several peptides across residues 226–250, including the Glu232/Arg236 region, remained comparatively protected. Thus, acidification does not globally destabilize the active-site region but selectively increases exchange in discrete structural elements adjacent to catalytic and ligand-binding residues.

Third, the C-terminal β-sheet core containing Arg320, Val325 and Asp326 showed heterogeneous pH sensitivity (Fig. 8c and Supplementary Fig. 14). Peptides spanning residues 318–334 exhibited relatively high baseline exchange with limited condition dependence; residues 335–348 showed modest acid sensitivity, and distal C-terminal peptides spanning residues 363–381 were largely pH- and ligand-invariant. Together, the MD and HDX-MS analyses show that acid- and ligand-sensitive exchange is localized to discrete dynamic modules rather than distributed uniformly across CK-BB.

### Ligand binding buffers global acid-induced deprotection while preserving local pH sensitivity

To compare apo and ligand-bound states directly, we mapped deuterium uptake differences at 60 s onto CK-BB structures and peptide-level bar plots (Fig. 9). In apo CK-BB, acidic pH broadly increases exchange across the NTDs, top convex surface and His191/Ser199-containing concave region (Figs. 9a, 9c, and 9e). Ligand binding substantially attenuates this global acid-induced deprotection, particularly across the otherwise dynamic convex and NTD regions (Figs. 9b, 9e, and 9h). For this direct comparison, the analysis was restricted to the 120 peptides that were detected and matched across both apo and ligand-bound datasets.

However, ligand binding did not uniformly suppress pH sensitivity. The His191/Ser199 region retained a localized acid response (Fig. 9b), and the short helix preceding Ser199 became more sensitive to graded pH changes in the ligand-bound state (shaded region, Figs. 9c–9e). Positive differences at pH 5.5 show that ligand binding protects large portions of CK-BB from acid-induced exchange (Fig. 9h), whereas localized negative or attenuated differences identify regions that remain dynamic or become selectively sensitized upon ligand binding (Fig. 9f–h). Thus, substrate binding stabilizes the global dimer while priming a regulatory concave-surface region for pH-dependent remodeling.

## Discussion

This study identifies progressive dimer opening as a novel regulatory mechanism by which protonation and substrate occupancy remodel human CK-BB and couple this soluble metabolic enzyme to dynamic membrane compartments. Using an integrated framework that combines cellular redistribution, DEER spectroscopy, HDX-MS, DEER-guided modeling, and long-timescale MD simulations, we show that CK-BB samples an acid- and substrate-regulated conformational landscape in which dimer opening is linked to region-specific remodeling of the convex and concave surfaces. Acidification redistributes CK-BB to punctate vesicular structures and membrane ruffles, increases backbone exchange across selected regions, and shifts the dimer toward more open conformations. This opening is coupled to lateral dimer expansion (PC1, Supplementary Video 1), with ligand-bound CK-BB at pH 5.5 occupying the most laterally expanded and open region of the conformational landscape. Substrate binding, in turn, buffers broad acid-induced deprotection while preserving and sharpening local pH sensitivity near the His191/Ser199 catalytic-regulatory interface. Together, these findings define dimer opening as a regulatory mechanism that generates membrane-competent CK-BB conformations and links local ATP regeneration to curved, actin-remodeling membrane domains.

A central insight is that CK-BB dynamics are spatially asymmetric. The convex face of the dimer, including the NTD interface and C-terminal β-sheet core, remains comparatively restrained according to DEER under most conditions, except under strongly acidic conditions; consistently, the top of this surface becomes HDX-sensitive upon acidification. Because HDX labeling times were pH-adjusted, the increased uptake observed under acidic conditions is best interpreted as a region-specific structural-dynamics response to acidification. Substrate binding further converts the threshold-like apo response into a more graded pH-sensitive transition.

The concave His191/Ser199 region behaves differently. DEER measurements of this surface reveal pH- and ligand-dependent redistribution among multiple conformational intermediates, whereas HDX-MS shows ligand-sensitized increases in local backbone exchange near His191 and Ser199. His191 lies within the nucleotide-binding environment, while Ser199 occupies a regulatory surface adjacent to a short helix connected to the CTD core. In the region preceding the phosphorylation site, highlighted by the gray shading and asterisk in Figs. 9c–9e, apo CK-BB displays a threshold-like acid response, whereas ligand-bound CK-BB shows a more graded pH-dependent increase in exchange. Thus, DEER captures large-scale inter-protomer remodeling of the concave surface, while HDX-MS resolves accompanying local changes in backbone dynamics and/or solvent exposure. Their convergence identifies the His191/Ser199 region as a ligand-sensitized, acid-responsive regulatory module through which active-site occupancy is communicated to dimer-scale opening. Notably, in the ligand-bound state, the region surrounding the phosphorylation site, which is coupled to the NTD–NTD interface, becomes more dynamic or exposed even under mildly acidic conditions (pH 6.5). This supports a potential regulatory mechanism that may facilitate phosphorylation of Ser199 by ERK1/2 MAPK or AMPK-linked stress-responsive pathways. Consistent with this possibility, AMPK acts as a cellular energy sensor that responds to metabolic stress and is additionally regulated by phosphagen balance, such that reduced PCr, increased creatine, and mild acidification can promote AMPK activation^71^.

The peptide-resolved HDX and MD data further refine this mechanism. The conserved flexible loop containing His66, Ile69 and Val72 is strongly acid-sensitive, consistent with prior structural and mutational work assigning this loop to active-site closure and catalytic organization^42,44,45^. Substrate binding extends this pH sensitivity to additional functional regions, including peptides containing Arg130, Arg132, Glu232, Arg236, Cys283, Arg292 and His296, indicating that ligand occupancy broadly tunes the pH responsiveness of catalytic and substrate-binding elements. At the same time, several peptides spanning the Glu232/Arg236 region and portions of the CTD core remain comparatively protected, showing that acidification does not globally destabilize CK-BB. Instead, acidic-pH selectively enhances exchange in discrete dynamic modules while preserving protected catalytic and core regions. This organization may allow CK-BB to maintain catalytic competence while increasing the conformational flexibility required for localization or membrane-proximal function.

These findings provide a structural rationale for the long-standing connection between CK-BB and actin-rich membrane compartments. Previous cell-biological studies showed that CK-B accumulates in membrane ruffles and phagocytic cups, where local CK activity supports ATP-dependent actin remodeling^9,10,26^. Our imaging data extend this framework by showing that acidification alone is sufficient to redistribute endogenous and recombinant CK-BB from a diffuse cytosolic pool to punctate vesicular structures and membrane ruffles. In parallel, the structural dynamics data suggest how a soluble enzyme lacking a canonical membrane anchor can nevertheless become enriched at such sites: acidification and substrate binding promote dimer opening, reshape dynamic convex and concave surfaces, and expose or stabilize conformations that may favor membrane-proximal retention.

Membrane curvature may provide an additional layer of regulation. Curvature-sensitive peripheral membrane proteins often use cationic, amphipathic, or concave surfaces to engage highly curved, negatively charged membranes. The crescent-like CK-BB dimer contains distinct convex and concave faces, raising the possibility that pH, substrate binding, and membrane curvature jointly tune those surfaces that engage vesicular or actin-remodeling membranes. Although the present data do not directly define the lipid-binding interface, they identify acid- and ligand-responsive surfaces that could plausibly mediate curvature-sensitive membrane association. This interpretation is consistent with acid-induced redistribution to vesicular and cortical compartments, while leaving direct secretion, membrane insertion, and translocation as important future tests.

Integrating these observations, we propose a model in which acidic pH and substrate binding remodel CK-BB to support membrane-proximal function (Fig. 10). In this model, progressive dimer opening functions as a regulatory switch that increases dynamics across the top convex surface and NTD–NTD interface, sensitizes the His191/Ser199-containing concave region, and generates membrane-competent CK-BB conformations. These changes may help the dimer adapt to curved vesicular and cortical membrane surfaces formed during ruffling, endocytic recycling or vesicle-associated trafficking. Locally recruited CK-BB could then regenerate ATP at sites of membrane remodeling, supporting actin polymerization, membrane protrusion, and vesicle-associated dynamics. In this framework, CK-BB is not simply a soluble ATP-buffering enzyme, but an acid- and substrate-regulated metabolic module whose conformational state tunes localization and coupling to dynamic membrane compartments.

These findings are relevant to cancer and stress physiology. Hypoxic and metabolically stressed tumor cells can release CK-BB, and extracellular CK-BB has been linked to phosphocreatine generation, anoikis resistance, hypoxic survival and metastatic progression^11,13,16^. More recent work connects creatine-phosphagen metabolism to mechanically driven pancreatic cancer invasion, SLC6A8-dependent metabolic vulnerabilities, glioblastoma growth in hypoxic niches, CK-dependent tumor-cell migration and invasion, and ferroptosis suppression through a moonlighting GPX4-phosphorylation function^18–23^. Because acidic pH is a common feature of hypoxic and metabolically active microenvironments, and local acidification accompanies endosomal, vesicular, and secretory pathways, a pH- and substrate-responsive CK-BB dimer may provide a molecular route by which metabolic stress promotes CK-BB relocalization, membrane association, and extracellular availability. The ability of substrate binding to buffer global deprotection while preserving localized dynamics may help maintain enzymatic competence during membrane engagement.

The strength of the current model lies in the convergence of orthogonal readouts, including DEER, HDX-MS, MD simulations, DEER-guided modeling, and cellular redistribution. Future studies will be needed to define the membrane-binding determinants, lipid specificity, curvature dependence, catalytic consequences and effects of phosphorylation at Ser6 and Ser199. Together, these results establish dimer opening as a novel regulatory mechanism that tunes CK-BB for local ATP buffering at dynamic membranes. This structural-dynamics framework connects catalytic regulation, vesicular redistribution and actin-remodeling microdomains, and provides a foundation for understanding how CK-BB contributes to cancer metabolism, extracellular phosphocreatine production, and stress-associated membrane remodeling.

## Methods

No statistical methods were used to predetermine sample size. Experiments were not randomized. Investigators were not blinded to allocation during experiments or outcome assessment.

### Cell culture, acidification and immunofluorescence

HCT116, HCT116/FLAG-CK-BB, HEK293 and HEK293/FLAG-CK-BB cells were cultured overnight on glass-bottom chambers coated with 2 μg cm^−2^ recombinant human laminin-521 (Gibco, A29248). Cells were treated for 1 h in Krebs–Ringer Bicarbonate HEPES buffer (KRBH) containing 129 mM NaCl, 4.8 mM KCl, 1.2 mM KH_2_PO_4_, 1.2 mM MgSO_4_, 2.5 mM CaCl_2_, 20 mM HEPES and NaHCO_3_ as indicated. Acute extracellular acidification was used as a stress-like perturbation to test CK-BB redistribution. Target pH values were obtained by directly titrating acidic solutions into KRBH before application to cells. To maintain pH during the 1-h treatment in a 5% CO_2_ incubator, neutral pH 7.2–7.4 medium was prepared in KRBH containing 20 mM NaHCO_3_, whereas acidic pH 5.5 medium was prepared in KRBH without NaHCO_3_, consistent with published guidelines^77^. Cells were fixed with 4% paraformaldehyde for 30 min at room temperature, permeabilized with 0.1% Triton X-100 in PBST, consisting of PBS containing 0.05% Tween 20, for 10 min at room temperature, and blocked for 1 h in PBST containing 1% BSA and 5% goat serum. Cells were incubated overnight at 4 °C with anti-CK-BB antibody (Proteintech, 15137–1-AP) or anti-FLAG antibody (Sigma, F3165) diluted in PBST containing 1% BSA, followed by 1 h incubation with goat anti-rabbit Alexa Fluor 488 or goat anti-mouse Alexa Fluor 594 secondary antibodies. Images were collected using a Zeiss LSM880 fluorescence microscope.

### Site-directed mutagenesis

The human CK-BB gene was cloned into the pET28b vector, which encodes an N-terminal 6-His tag under the control of an inducible T7 promoter. The five cysteine residues in CK-BB were mutated (C74A, C141T, C146S, C254S and C283D) by site-directed mutagenesis using complementary oligonucleotide primers, yielding the CL protein. This construct was used as the template to introduce single cysteines for the DEER constructs. Substitution mutations were generated using a single-step PCR in which the entire template plasmid was replicated from a single mutagenic primer. CK-BB mutants were sequenced using both T7 forward and reverse primers to confirm mutagenesis and the absence of aberrant changes. Mutants were identified by the native residue and primary sequence position followed by the substituted residue.

### Expression, purification, and spin labeling of CK-BB

*Escherichia coli* BL21 (DE3) cells were freshly transformed with pET28b vector encoding WT or recombinant CK-BB mutants. A transformant colony was used to inoculate Luria–Bertani (LB) media (Fisher Bioreagents) containing 0.05 mg/mL kanamycin (Gold Biotechnology), which was grown overnight (~15 h) at 37 °C and was subsequently used to inoculate 3–6 L of LB at a 1:50 dilution. Cultures were incubated while being shaken at 37 °C until they reached an absorbance at 600 nm (Abs_600nm_) of ~ 0.5–0.7, at which time CK-BB expression was induced by the addition of 0.25 mM IPTG (Gold Biotechnology). The cultures were incubated for 4 h at 30 °C and then harvested by centrifugation. Cell pellets were resuspended in lysis buffer (20 mM HEPES, pH 7.5, 300 mM NaCl, and 5% [vol/vol] glycerol) at 25 mL per liter of culture, including 2 mM DTT, and 1 mM PMSF, and the cell suspension was lysed via sonication for an 8-min total elapsed time using 15-s on/15-s off pulses at 30% amplitude. Cell debris was removed by centrifugation at 30,000 r.p.m. (105,000 × *g*) for 30 min at 4 °C in a Type 45 Ti rotor (Beckman Coulter). The supernatant was incubated with 2.0 mL (bed volume) His60 Ni Superflow resin (TakaraBio) for 2 h at 4 °C, washed with 10 column volumes of buffer containing 30 mM imidazole and eluted with buffer containing 300 mM imidazole. The eluate was desalted into PBS containing 5% glycerol by using a Cytiva HiTrap desalting column. The His tag was removed by thrombin protease (MP Biochemicals) overnight at 4 °C at a 1:1000 thrombin:CK-BB mass ratio (ε_CK-BB-CL_ = 35,410 M^−1^·cm^−1^, M.W. ~ 44,753 Da). For DEER spectroscopy, single-cysteine mutants were labeled with one round of 10-fold molar excess 1-oxyl-2,2,5,5-tetramethylpyrroline-3-methyl methanethiosulfonate (Enzo Life Sciences) per cysteine. Thrombin was removed using a Cytiva HiTrap Heparin HP column (20 mM Tris pH 7.5, 5% glycerol), and cleaved CK-BB was separated from uncleaved protein and free His tag by using 2 mL Ni resin on a gravity flow column while collecting cleaved CK-BB in the flow-through. Final purification was performed by size-exclusion chromatography on a Superdex 200 Increase 10/300 GL column (Cytiva) equilibrated in 50 mM Tris/MES, pH 7.3, 100 mM NaCl buffer, with or without 10% (vol/vol) glycerol buffer for DEER spectroscopy and MS analyses, respectively. Peak fractions of purified CK-BB were combined and concentrated with 30,000 MWCO Amicon ultra filter concentrator (Millipore, Sigma-Aldrich). The final concentration was determined by A_280_ measurement (ε = 35,410 M^−1^·cm^−1^, M.W. ~ 42,871 Da) for use in subsequent studies. WT sample was submitted to native and denaturing MS analyses to confirm that the measured molecular weight agreed with that from the amino acid sequence.

### Creatine kinase activity assay

Creatine kinase activity was measured using the creatine kinase activity kit (MAK116, Sigma-Aldrich). Recombinant CK-BB was diluted to 0.1 mg/mL in 50 mM Tris, pH 7.5, and 1 μg protein was used per reaction. Reactions were initiated by adding reconstituted kit reagent containing assay buffer, enzyme mix, and substrate solution. For pH-dependent assays, the reagent was prepared in 50 mM Tris/MES and 150 mM NaCl buffers adjusted to the indicated final pH values. Absorbance at 340 nm was monitored every minute for 40 min at 37 °C on a SpectraMax i3 plate reader. Activity was determined from the linear change in NADH-coupled absorbance according to the manufacturer’s equation. Experiments were performed at least in triplicate, and mean ± s.d. values were calculated.

### Circular dichroism spectroscopy

Far-UV circular dichroism spectra were recorded for WT and CL CK-BB, and for spin-labeled mutants. Proteins were diluted to 0.01 mg/mL in 10 mM sodium phosphate, pH 7.3. Spectra were collected from 195 to 260 nm at 22 °C using an Applied Photophysics Chirascan V100 in a 10-mm quartz cell. Data were collected at 1-nm intervals with 0.1 s per point and 10 averaged scans. Experiments for WT and CL CK-BB were performed in triplicate, and mean ± s.d. values were calculated.

### CW-EPR and DEER spectroscopy

Room-temperature continuous-wave (CW) EPR spectra of spin-labeled CK-BB samples were collected to assess labeling quality. CW-EPR spectra were collected at room temperature on a Bruker EMX spectrometer operating at X-band frequency (9.5 GHz) using 10-mW incident power and a modulation amplitude of 1.6 G. DEER spectroscopy was performed on an Elexsys E580 EPR spectrometer operating at Q-band frequency (33.9 GHz) with the dead-time free four-pulse sequence at 83 K^78^. Pulse lengths were 20 ns (π/2) and 40 ns (π) for the probe pulses and 40 ns for the pump pulse. The frequency separation was 63 MHz. To ascertain the role of H^+^, samples were titrated to pH 6.5 and 5.5 with empirically determined amounts of HCl and confirmed by pH microelectrode (Mettler Toledo InLab Ultra-Micro-ISM) measurement. The substrate-bound state was generated by addition of 10 mM MgATP, 0.8 mM ADP, 20 mM PCr and 10 mM Cr. Samples for DEER analysis were cryoprotected with 24% (vol/vol) glycerol and flash-frozen in liquid nitrogen. Primary DEER decays were analyzed using home-written software operating in the Matlab (MathWorks) environment as previously described^79^. Briefly, the software carries out global analysis of the DEER decays obtained under different conditions for the same spin-labeled pair. The distance distribution was assumed to consist of a sum of Gaussians, the number and population of which were determined based on a statistical criterion. The generated confidence bands were determined from calculated uncertainties of the fit parameters. We also analyzed DEER decays individually and found that the resulting distributions agree with those obtained from global analysis. Comparison of the experimental distance distributions with the CK-BB models and structures using a rotamer library approach was facilitated by the MMM software package^80^. Rotamer library calculations were conducted at 175 K.

### DEER-guided AlphaFold modeling

DEER-guided modeling of CK-BB was performed using DEERFold^53^, a fine-tuned AlphaFold2-based^55^ network that incorporates experimental DEER distance distributions as pairwise distograms within the Evoformer module, together with the multiple sequence alignment (MSA), to bias structure prediction toward spin-label-constrained conformations. The DEERFold architecture, training strategy, and input-encoding scheme were described previously^53^ and were used here without modification.

The CK-BB amino acid sequence was used as input, and its MSA was generated with the ColabFold MSA pipeline^81^. For DEERFold predictions, the MSA was subsampled to an effective sequence depth of *N*_eff_ = 10^82^. For each restraint condition described below, 50 independent models were generated using distinct random seeds, producing a conformational ensemble for that condition. Models were ranked by the Earth Mover’s Distance (EMD) between the predicted and experimental distance distributions, and the highest-ranked subset, hereafter referred to as the “top models,” was retained for downstream structural analysis.

For each spin pair, single-Gaussian approximation, defined by its mean distance and standard deviation, of the stabilized distance component observed under each condition was used as DEERFold restraints. Each single-Gaussian restraint set was supplied to DEERFold as an independent input, generating separate ensembles. Principal component analysis was performed on refined ensembles to identify dominant collective motions. PC1 reports lateral dimer expansion, whereas PC2 reports hinge-like opening and closure of the dimer.

### DEER-guided refinement of the unbiased AlphaFold3 models

Unbiased models of CK-BB in the apo and MgATP-bound states were generated using AlphaFold3 (AF3)^56^, as implemented in the AF3 server. Five independent runs were performed for each state, and the highest-pLDDT model was selected as the reference conformation. The DEER distance constraints were used to refine the AF3-generated models using a previously published approach^54^. Refinement was carried out iteratively in MODELLER^83^. In silico spin labeling was performed with the MMM software package^80^ using a rotamer-library approach. In each iteration, rotamer ensembles were first calculated at 298 K for the spin-labeled sites, and the rotamer that best matched the mean N–O midpoint position of the full ensemble was attached to the template structure provided to MODELLER. Refinement was performed using the centers of the Gaussian components corresponding to each conformational state, together with secondary-structure restraints derived from AlphaFold models. All refined models achieved a GA341 score of 1.0. After import into MMM, rotamer ensembles were recalculated (Supplementary Figs. 4 and 5), and models were ranked by the root-mean-square deviation between the experimental distance constraints and the corresponding model-derived distances across all restraints.

### Hydrogen-deuterium exchange mass spectrometry

Bottom-up HDX-MS experiments were performed following established peptide-level HDX-MS principles^72–75^. Briefly, 5 μL of 20 μM CK-BB was diluted into 95 μL deuterated labeling buffer containing 25.6 mM Tris, 24.4 mM MES and 100 mM NaCl at the indicated pH values. Samples were labeled in triplicate at 20 °C for each pH-adjusted labeling time. Exchange was quenched by adding 100 μL quench buffer containing 100 mM glycine and 4 M urea, pH 2.4, precooled to 0 °C. Nondeuterated controls were prepared identically using H_2_O-based labeling buffer. For deuterated labeling buffers, pD was calculated from the apparent pH as pD = apparent pH + 0.4. For each labeling condition, the final quenched protein–buffer mixture was measured, and the quench composition was adjusted as needed to ensure equivalent final quench pH across all conditions. HDX-MS measurements were performed in triplicate at each labeling time and experimental condition (*n* = 3 technical replicates). Data are presented as the mean ± s.d., where applicable.

After quenching, 200 μL of each sample was injected using a fully automated Trajan LEAP robot and passed through a custom-prepared immobilized pepsin column (2.1 × 50 mm) at 0.2 mL min^−1^ using an Agilent 1290 LC system. The resulting peptic peptides were trapped and desalted on a ZORBAX 300SB-C8 trap column (2.1 × 12.5 mm) and separated on an XSelect CSH C18 column (2.1 × 50 mm, 2.5 μm; Waters) using a linear 14-min gradient from 5% to 100% solvent B. Solvent A was 100% H_2_O containing 0.1% formic acid, and solvent B was 80:20 acetonitrile:H_2_O containing 0.1% formic acid. Peptide *m/z* values were measured on a Bruker Maxis II Q-TOF ETD mass spectrometer. Raw data were analyzed using HDExaminer v3.4.1, exported directly from HDExaminer,and submitted to Kingfisher^57^ for downstream statistical analysis and visualization.

### pH correction and HDX calculations

Because intrinsic backbone amide exchange slows at lower pH in the base-catalyzed regime, labeling times were adjusted to separate intrinsic pH effects on exchange chemistry from condition-dependent changes in protein dynamics or solvent exposure. The pH correction factor was calculated relative to the reference condition, pH 7.3, using Equation (1):

(1) $$F_{\text{pH}}=10^{\left({pH}_{ref}-pH\right)}$$

where pH_ref_ = 7.3. A reference labeling time at pH 7.3 was converted to the matched lower-pH labeling time using Equation (2):

(2) $$t_{pH}=t_{7.3}\times F_{pH}$$

This procedure yielded correction factors of 6.3, 20.0 and 63.1 at pH 6.5, 6.0 and 5.5, respectively (Supplementary Table 1). These extended labeling times allowed deuterium uptake to be compared across pH conditions on matched intrinsic-exchange windows. Peptide centroid masses were calculated from the isotope envelope using Equation (3):

(3) $$m_{\text{centroid}}=\frac{\sum_{i}I_{i}m_{i}}{\sum_{i}I_{i}}$$

where *Ii* and *mi* are the intensity and mass of isotope peak *i*. Deuterium uptake was calculated from the centroid mass after labeling (*mt*), the undeuterated centroid (*m*_0_) and the fully deuterated or maximum-exchange reference (*m*_100_) using Equation (4):

(4) $$\%D(t)=100\times \frac{m_{t}-m_{0}}{m_{100}-m_{0}}$$

Pairwise deuterium differences were calculated for each comparison using Equation (5):

(5) $$\Delta \%D_{A-B}=\%D_{A}-\%D_{B}$$

Positive values indicated greater exchange in condition A relative to condition B. Together, the pH-adjusted exchange times, centroid-based uptake calculations, and peptide-level comparisons enabled condition-dependent changes in local backbone dynamics and/or solvent exposure to be distinguished from intrinsic pH-dependent changes in amide exchange kinetics.

### Peptide identification and HDX data analysis

Peptide identification was performed by LC-MS/MS in data-dependent acquisition mode on a Bruker Maxis II Q-TOF ETD mass spectrometer prior to conducting HDX. MS/MS spectra were searched and curated in Protein Metrics software using a score cutoff of 200 and manual validation of fragment assignments. HDX data were analyzed with Kingfisher^57^ using a hybrid significance-testing approach that combines a peptide-level statistical test with a global experimental-error threshold^84,85^. All peptide isotope envelopes used in the differential HDX-MS analysis were manually inspected for EX1-type bimodal isotope distributions, and no evidence of EX1 exchange was observed.

For each pairwise comparison, Welch’s *t* statistic was calculated using Equation (6):

(6) $$t=\frac{{\overset{‾}{x}}_{A}-{\overset{‾}{x}}_{B}}{\sqrt{s_{A}^{2}/n_{A}+s_{B}^{2}/n_{B}}}$$

with Welch-Satterthwaite degrees of freedom calculated using Equation (7):

(7) $$v=\frac{{\left(s_{A}^{2}/n_{A}+s_{B}^{2}/n_{B}\right)}^{2}}{\frac{{\left(s_{A}^{2}/n_{A}\right)}^{2}}{n_{A}-1}+\frac{{\left(s_{B}^{2}/n_{B}\right)}^{2}}{n_{B}-1}}$$

To estimate the magnitude threshold required for significance, a pooled standard deviation was calculated across replicate measurements using Equation (8):

(8) $$s_{p}=\sqrt{\frac{\sum_{j}\left(n_{j}-1\right)s_{j}^{2}}{\sum_{j}\left(n_{j}-1\right)}}$$

The propagated standard error for a difference between two states was then calculated using Equation (9):

(9) $${SEM}_{\Delta }=s_{p}\sqrt{\frac{1}{n_{A}}+\frac{1}{n_{B}}}$$

where *n*_*A*_ and *n*_*B*_ denote the numbers of experimental replicates for protein states A and B, respectively. The global significance threshold was calculated using Equation (10):

(10) $$GSE=t_{\alpha ,v}\times {SEM}_{\Delta }$$

where *tα, ν* is the critical value from Student’s *t*-distribution, selected according to the predefined significance level (α), and the applicable degrees of freedom (ν). A peptide was considered significantly changed only when the absolute deuterium difference exceeded the global significance threshold, and the Welch *t* test met the indicated p-value cutoff. Peptides meeting *p* < 0.001 were displayed as stringent hits, peptides meeting *p* < 0.05 were displayed as less stringent hits, and peptides failing either criterion were considered as showing a not significant difference. A peptide was classified as significant if at least one time point satisfied both criteria.

### Native and denaturing mass spectrometry

Native MS was performed under soft ionization and transmission conditions optimized to preserve noncovalent CK-BB dimers^86,87^. Spray voltages were maintained between 0.6 and 1.2 kV, and collision energies were adjusted as needed. Denaturing ESI-MS was performed on a Bruker MaXis II Q-TOF mass spectrometer. Samples were trapped on a ZORBAX 300SB-C3 column, desalted with water/acetonitrile/formic acid and eluted with a short acetonitrile gradient. Protein mass spectra were deconvoluted using Intact or UniDec.

### Molecular dynamics simulations

All-atom MD simulations were performed for four CK-BB states: apo pH 5.5, apo pH 7.3, ligand-bound pH 5.5 and ligand-bound pH 7.3. Starting structures were derived from AlphaFold3 models^56^. For the pH 5.5 systems, protonation states were assigned using PROPKA^88^ and applied symmetrically to both protomers. Protonated residues included Asp62, Glu19, Glu80, Glu155, Glu231, His0 and His66. No additional protonation was applied to the pH 7.3 systems. Systems were prepared in explicit solvent using CHARMM-GUI^89^, solvated with TIP3P water^90^, neutralized with Na^+^ and Cl^−^ ions, and supplemented with 0.15 M NaCl. The CHARMM36m force field was used^91^. Each solvated system contained approximately 179,000 atoms.

Initial energy minimization was performed for 10,000 conjugate-gradient steps. Systems were then equilibrated for approximately 19 ns in NAMD^92^ using the standard CHARMM-GUI restrained protocol in the NVT ensemble at 310 K, with a Langevin thermostat damping coefficient of 1.0 ps^−1^. A subsequent 15-ns pre-production run was performed in the NPT ensemble at 310 K and 1 atm, with pressure maintained using the Nosé–Hoover Langevin piston method^93^ and a 2-fs integration step. Equilibrated systems were then converted to Anton-compatible format, and triplicate 1-μs production simulations were performed on the Anton 3 supercomputer^94^ in the NPT ensemble using a Desmond-compatible simulation framework. Temperature was maintained at 310 K using an antithetic thermostat with an interval of 48 timesteps, and pressure was maintained isotropically at 1.0 bar using a Monte Carlo barostat with an interval of 480 timesteps. A 2.5-fs integration step with RESPA multiple time stepping was used. Trajectories were analyzed for RMSD, RMSF, interdomain angles and residue-specific dynamics using custom scripts and VMD^95^. RMSD was calculated relative to the first frame of each production trajectory after least-squares superposition of protein Cα atoms. For angle analysis, the relative orientation between equivalent regions of adjacent protomers was quantified from MD trajectories using custom Tcl scripts in VMD. Each frame was aligned to the initial structure by least-squares fitting of protein Cα atoms to remove global translation and rotation. For each protomer, the region of interest was defined by Cα atoms from residues 216–220, 225–229 and 235–242, and its orientation was defined by the principal axis of the Cα inertia tensor corresponding to maximal spatial extension. The inter-protomer angle was calculated from the dot product of the normalized principal axes and converted to degrees. To remove sign ambiguity, angles greater than 90° were mapped to their supplementary values, yielding a 0–90° measure of relative domain opening and closure between protomers.

## Supplementary Material

Supplementary Files

This is a list of supplementary files associated with this preprint. Click to download.

- SupplementaryVideo1.mpg
- SupplementaryVideo2.mpg
- SupplementaryInformation.pdf

## Figures and Tables

**Figure 1.**
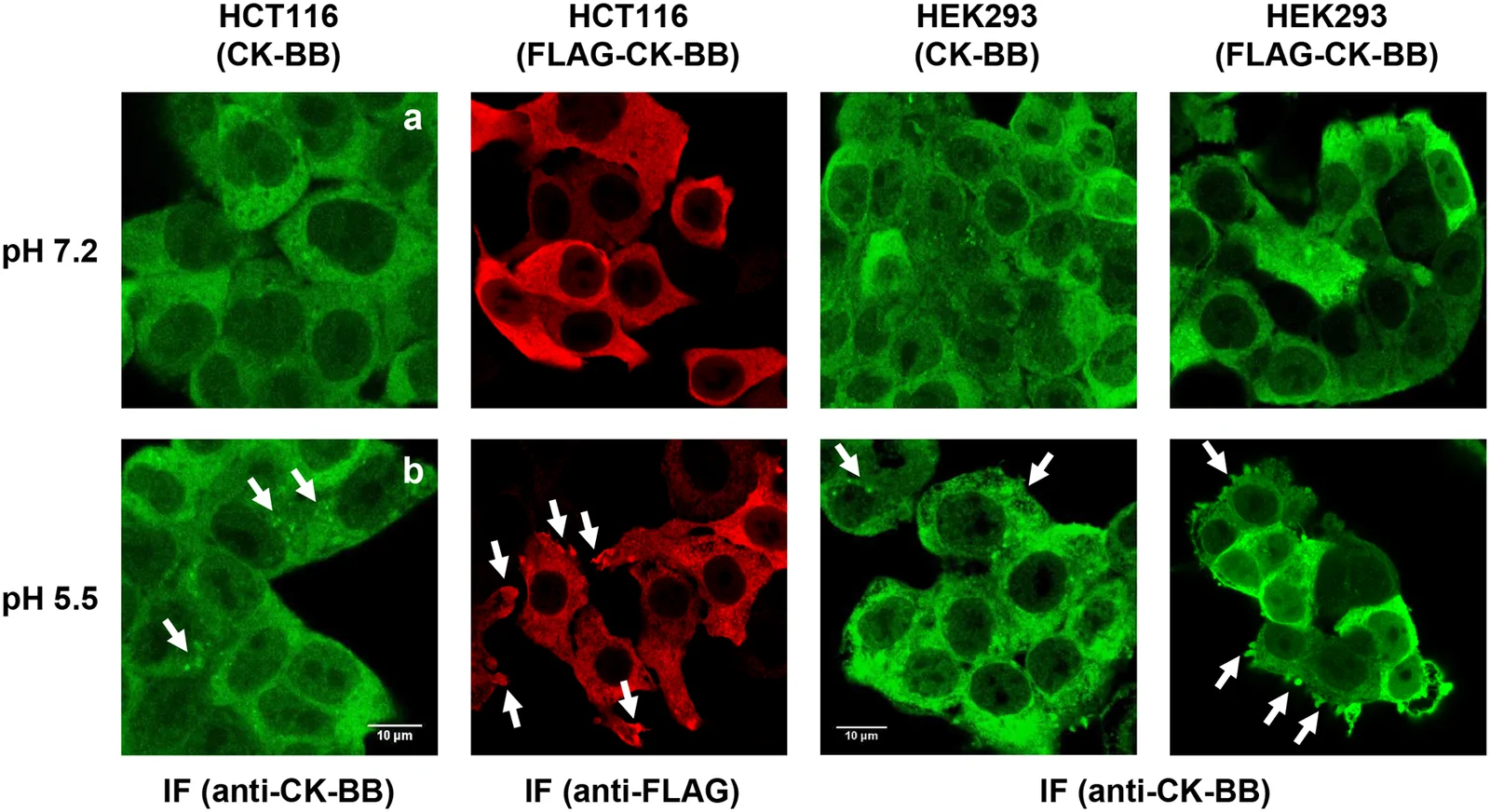
Acidic conditions redis tribute CK-BB to vesicles and membrane ruffles. Immunofluorescence staining of HCT116 colorectal carcinoma and HEK293 cells expressing endogenous CK-BB or recombinant FLAG-tagged CK-BB after incubation at pH 7.2 (**a**) or pH 5.5 (**b**) in a 5% CO_2_ incubator. Endogenous CK-BB was detected with an anti-CK-BB antibody (green), while recombinant FLAG-CK-BB was detected with anti-FLAG (red) or anti-CK-BB antibodies, as indicated. Compared with neutral pH, acidic conditions increased punctate/vesicular staining and enriched CK-BB at membrane ruffles. Scale bars, 10 μm.

**Figure 2.**
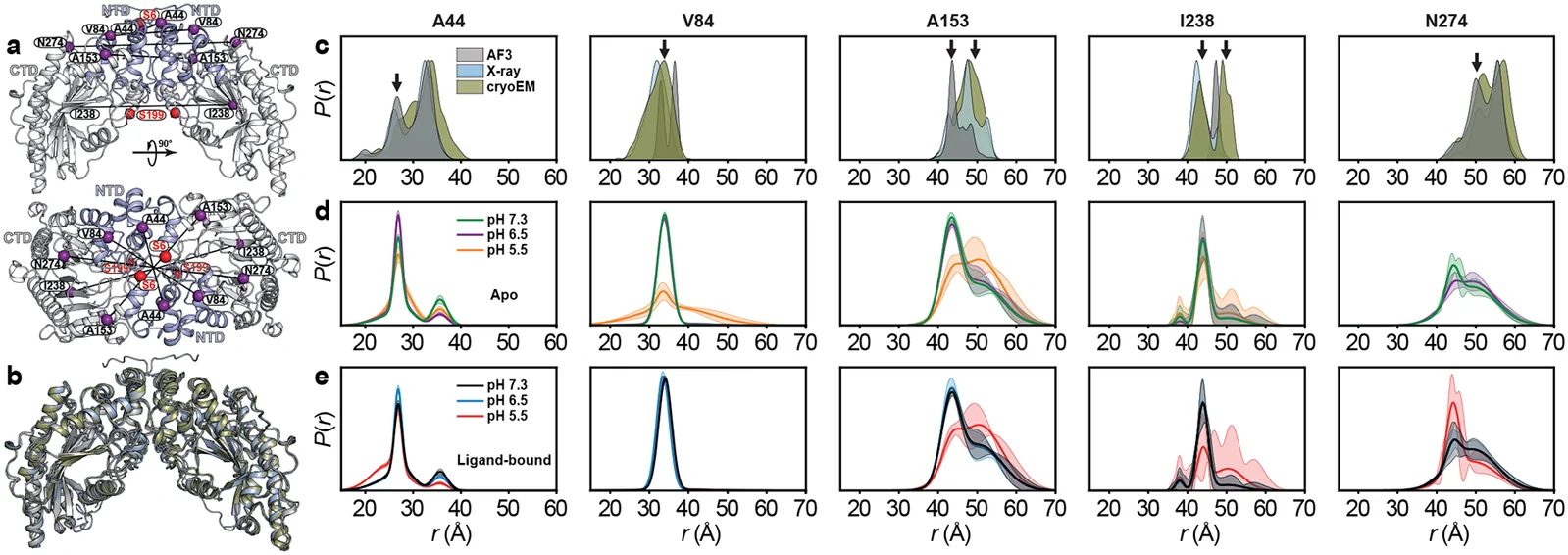
Conformational dynamics of the CK-BB dimer at the convex side of the NTD interface and CTD beta-sheet core. (**a**) Inter-protomer spin-label pairs (purple spheres) used for DEER measurements across the convex side of the NTD dimer interface and the C-terminal beta-sheet core. Putative phosphorylation sites Ser6 and Ser199 are shown as red spheres; NTD and CTD regions are colored light blue and grey, respectively. (**b**) Overlay of cryo-EM (green; PDB 6V9H), X-ray (blue; PDB 7TUN) and AlphaFold (gray) CK-BB structures. (**c**) Predicted spin-label distance distributions from the structures, with arrows marking components corresponding to experimentally observed DEER intermediates. (**d**,**e**) DEER distance distributions, *P*(*r*), for apo (**d**) and ligand-bound (**e**) CK-BB at physiological and acidic pH. Except under strongly acidic conditions (pH 5.5), these CK-BB regions remain relatively rigid and show minimal pH- or ligand-dependent changes. Confidence bands indicate 2σ uncertainty from fitting of the primary DEER traces. Raw DEER decays and fits are shown in Supplementary Fig. 2.

**Figure 3.**
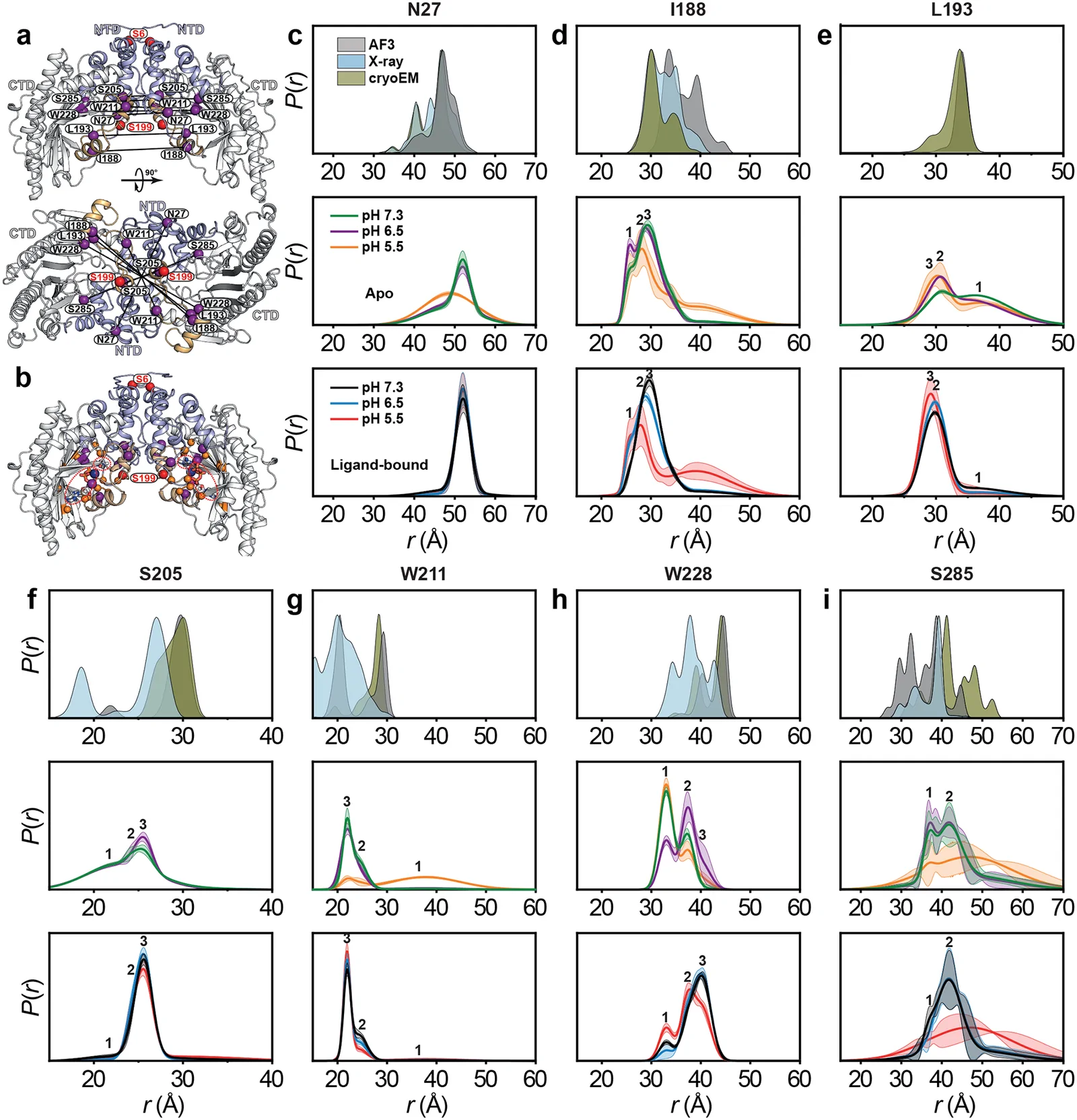
The dynamic concave side of the CK-BB dimer undergoes pH- and ligand-dependent conformational remodeling. (**a**) Inter-protomer spin-label pairs (purple spheres) used for DEER measurements across the concave side of the CK-BB dimer. Ser6 and Ser199 phosphorylation sites are shown as red spheres; NTD and CTD regions are colored light blue and gray, respectively, and the region flanking Ser199 is highlighted in light orange. (**b**) AlphaFold3 model of CK-BB bound to Mg^2+^ATP and creatine, with catalytic and substrate-binding residues shown as orange spheres. (**c–i**) Predicted spin-label distance distributions from AF3, X-ray and cryo-EM structures (top) are compared with DEER-derived distance distributions, *P*(*r*), for apo (middle) and ligand-bound CK-BB (bottom) at physiological and acidic pH. The concave side, particularly the Ser199-containing region, is highly dynamic, displays pH- and ligand-dependent conformational changes, and samples three distinct conformational intermediates. Confidence bands indicate 2σ uncertainty from fitting of the primary DEER traces. Raw DEER decays and fits are shown in Supplementary Fig. 3.

**Figure 4.**
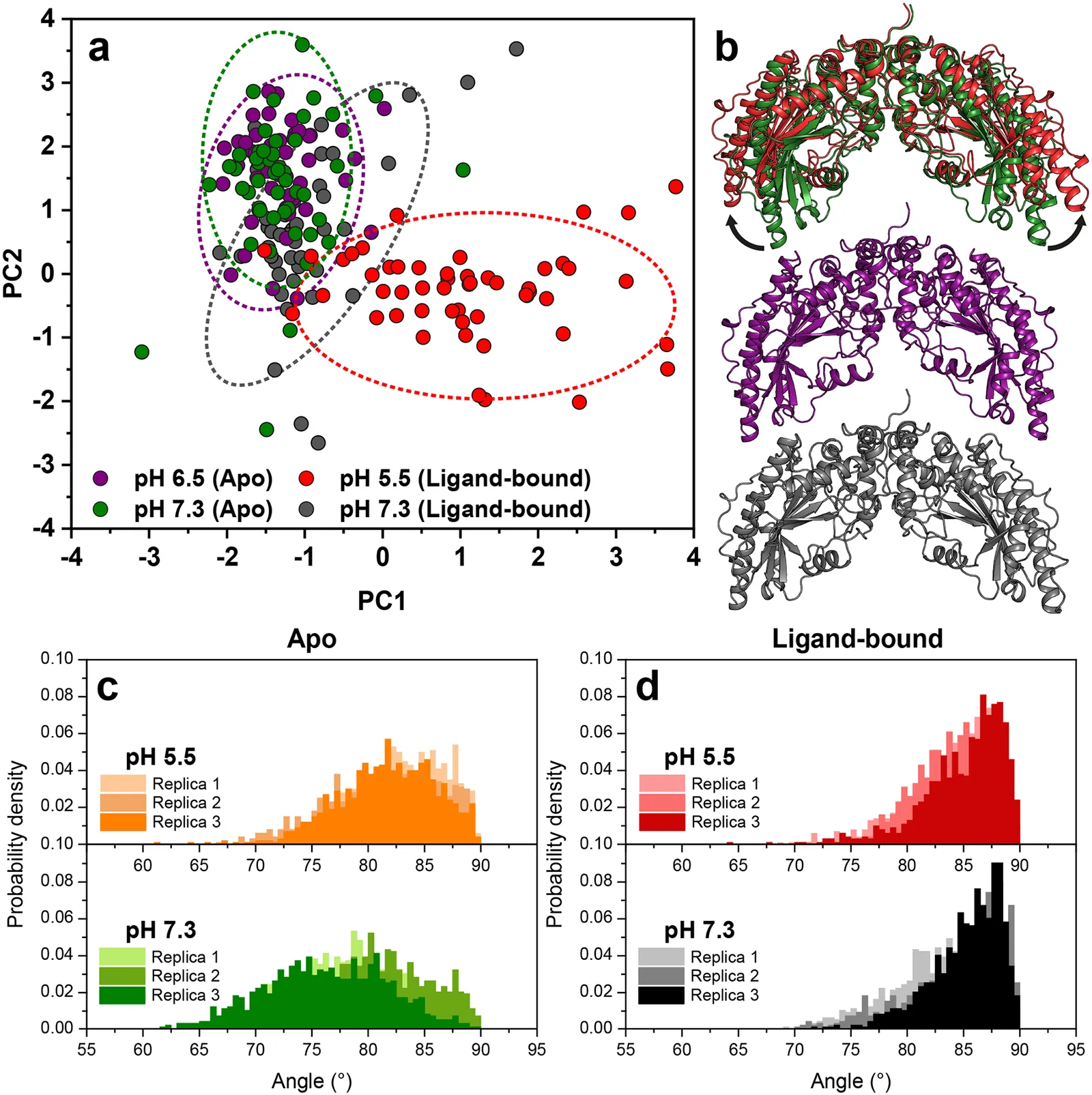
Acidic pH and ligand binding promote opening of the CK-BB dimer. (**a**) Principal component analysis of DEER-guided AlphaFold models. PC1 captures lateral dimer expansion, whereas PC2 reports hinge-like opening and closure of the CK-BB dimer (Supplementary Videos 1 and 2). Ligand-bound CK-BB at pH 5.5 occupies the most expanded and open conformational space, while ligand-bound CK-BB at pH 7.3 shows an intermediate distribution and apo states remain comparatively closed. (**b**) DEER-refined AF3 models of apo and ligand-bound CK-BB under physiological and acidic conditions (Supplementary Figs. 4 and 5), showing a transition from a more closed, dynamic apo ensemble at pH 7.3 to a more open ligand-bound conformation under acidic conditions. (**c**,**d**) Dimer-opening angle distributions from triplicate 1-μs MD simulations of apo (**c**) and ligand-bound (**d**) CK-BB at physiological and acidic pH. The MD simulations reproduce the ligand- and pH-dependent opening observed in the DEER-guided structural ensembles.

**Figure 5.**
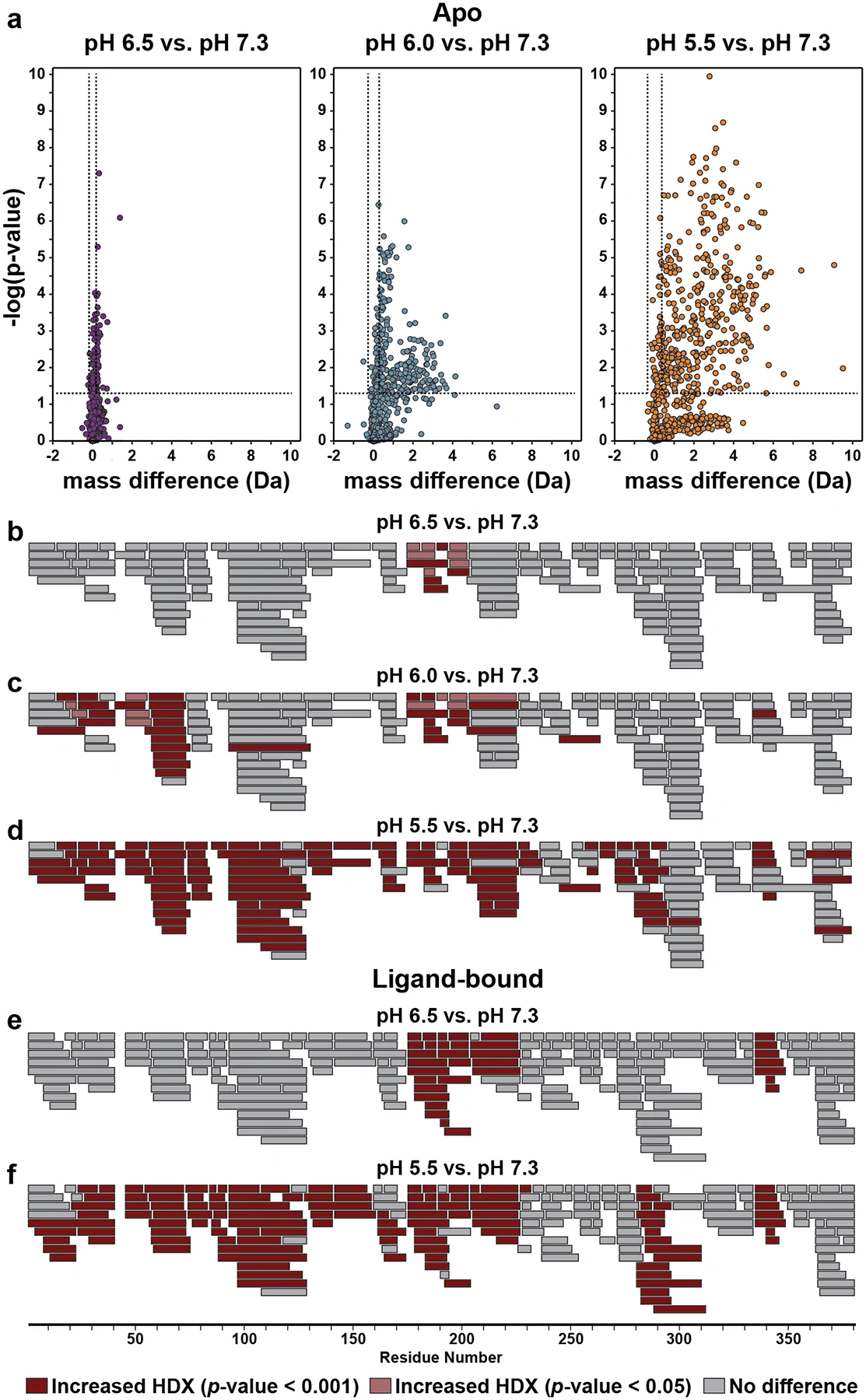
HDX-MS reveals enhanced CK-BB backbone dynamics under acidic conditions. (**a**) Volcano plots showing pH-dependent changes in deuterium uptake for apo CK-BB relative to pH 7.3. The vertical axis shows statistical significance (−log_10_ *p* value from *t* tests), with higher values indicating more significant changes; data points above the dotted threshold lines were considered significant. Lower pH progressively increases both the magnitude and significance of deuterium uptake, indicating enhanced conformational dynamics. (**b–f**) Peptide coverage maps for apo (**b–d**) and ligand-bound (**e**,**f**) CK-BB, showing regions with significantly increased HDX at the indicated pH values relative to pH 7.3. Dark red and light red indicate peptides meeting stringent (*p* < 0.001) and less stringent (*p* < 0.05) significance thresholds, respectively; gray indicates peptides that failed the hybrid significance test, either by not exceeding the global error threshold or by not meeting the minimum *p*-value cutoff.

**Figure 6.**
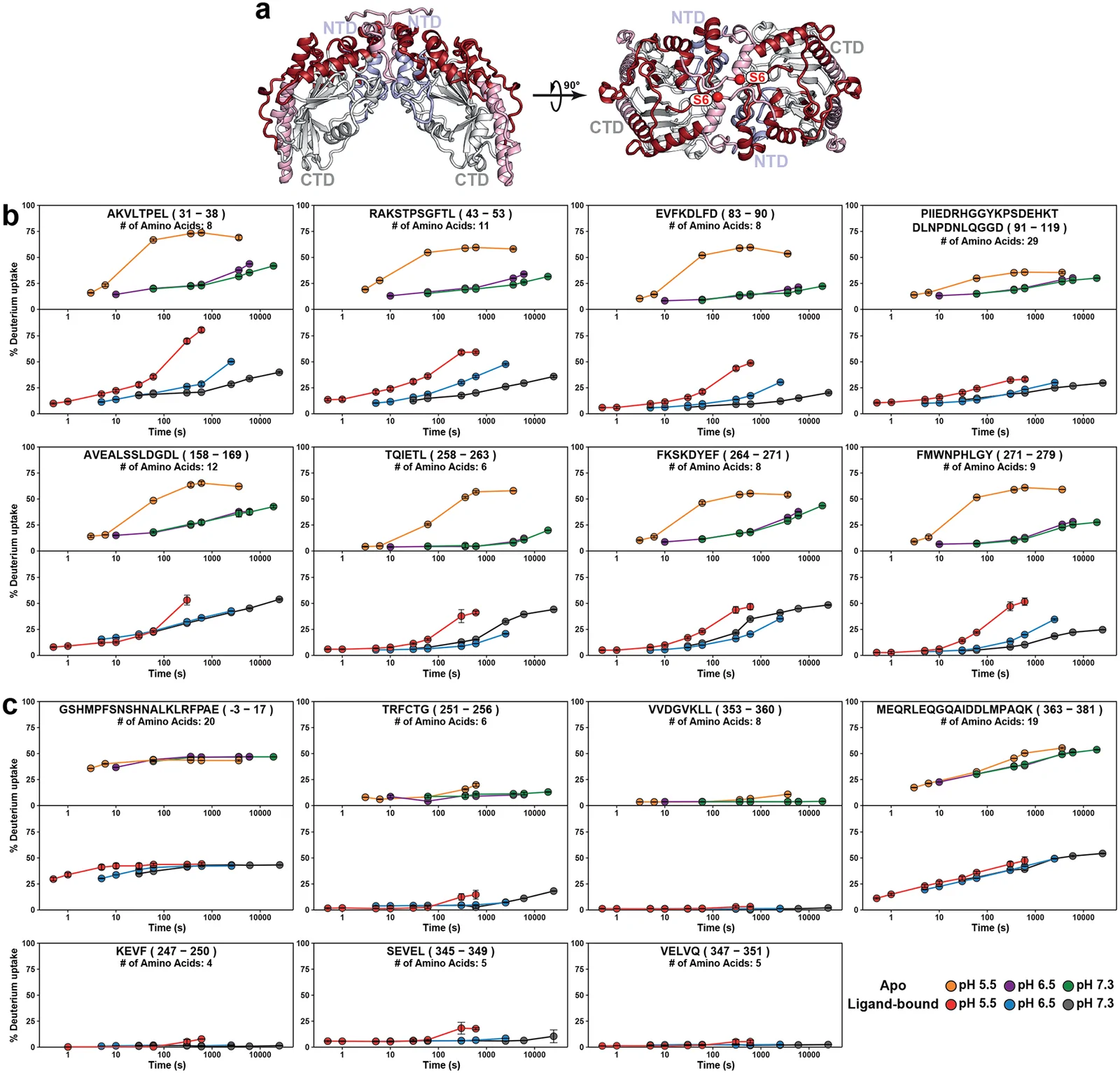
Acidic pH and ligand binding selectively enhance dynamics at the top of the CK-BB convex surface. (**a**) CK-BB dimer structure with convex-surface peptides mapped onto the NTD and CTD. Peptides showing increased deuterium uptake under acidic conditions are shown in dark red, whereas pH-invariant or minimally exchanging peptides are shown in light red; the Ser6 phosphorylation site is shown as a red sphere. (**b**) HDX kinetic plots for peptides near the top of the convex surface, excluding the N-terminal region, show pronounced pH-dependent increases in backbone exchange. In apo CK-BB, exchange is largely similar at pH 7.3 and 6.5 but increases markedly at pH 5.5, consistent with a threshold-like acid-induced transition. Ligand binding sensitizes this region to pH, producing modest but graded increases in HDX across all three pH conditions, consistent with allosteric, substrate-dependent priming of local backbone dynamics and/or solvent exposure. (**c**) In contrast, peptides along the lateral sides of the convex surface show minimal pH- or ligand-dependent changes, indicating comparatively stable or protected regions. Deuterium uptake is plotted as percentage uptake over exchange time for apo and ligand-bound CK-BB at pH 7.3, 6.5 and 5.5.

**Figure 7.**
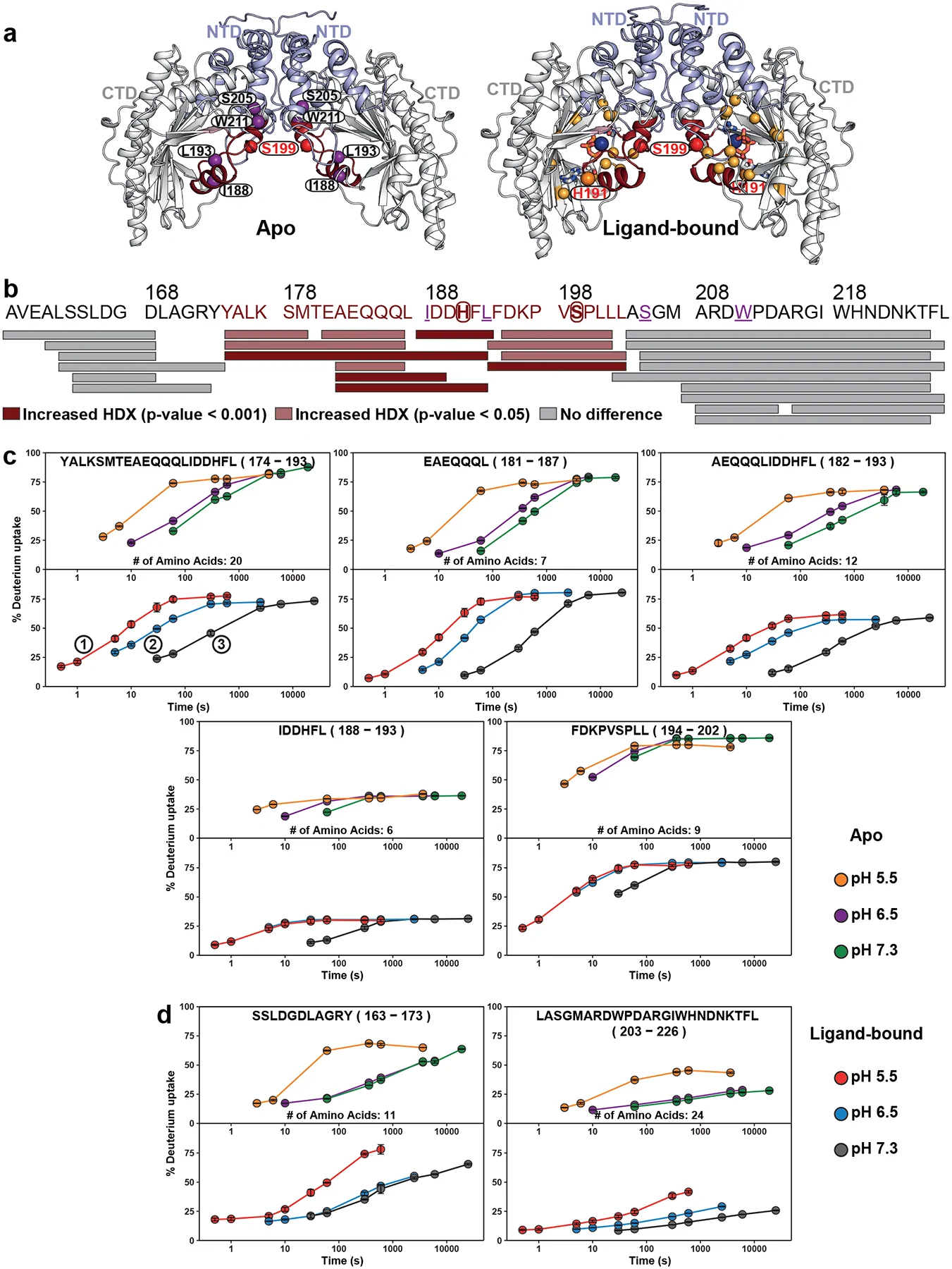
Ligand-sensitized, pH-dependent dynamics near His191 and Ser199 on the CK-BB concave surface. (**a**) Apo and ligand-bound CK-BB dimer models highlighting the concave-surface region spanning the nucleotide ribose-interacting residue His191, the putative Ser199 phosphorylation site, and associated DEER reporter positions (purple spheres, Fig. 3). Catalytic and substrate-binding residues are shown as orange spheres. (**b**) Expanded peptide coverage map for residues 158–226, highlighting peptides with significant pH-dependent increases in deuterium uptake. (**c**) HDX uptake plots for peptides spanning the His191/Ser199 region. In apo CK-BB, exchange at pH 6.5 remains closer to pH 7.3, whereas pH 5.5 markedly increases exchange, consistent with a threshold-like acid-induced enhancement of local backbone dynamics and/or solvent exposure. Ligand binding sensitizes this region to pH, producing graded increases in HDX across all three pH conditions, consistent with substrate-dependent priming of local dynamics. (**d**) HDX uptake plots for peptides flanking the His191/Ser199 region. Unlike the central region, these flanking peptides show primarily threshold-like acid-induced increases in exchange under both apo and ligand-bound conditions. Global significance error, 0.32 Da; *p* < 0.001.

**Figure 8.**
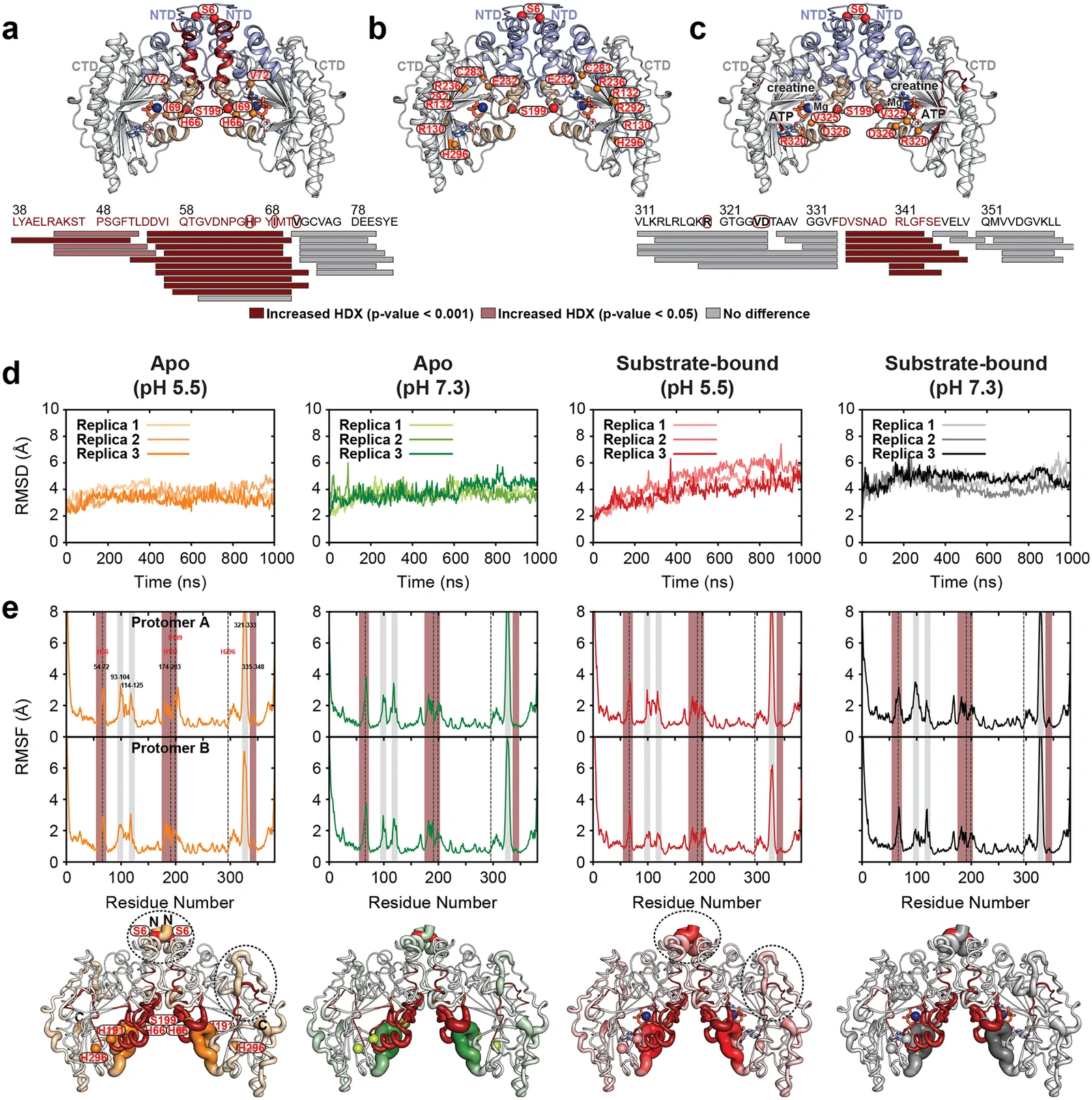
Molecular dynamics simulations link acid- and ligand-sensitive HDX regions to dynamic CK-BB structural elements. (**a**) CK-BB model highlighting the NTD interface and adjacent conserved flexible loop (residues 60–70), including His66 and Ile69, which show increased deuterium uptake under acidic conditions (Supplementary Fig. 8). Functional residues in this region are shown as orange spheres. (**b**) Additional catalytic and substrate-binding residues on the concave surface. (**c**) CK-BB model highlighting a C-terminal beta-sheet core region that shows increased deuterium uptake in the ligand-bound state under acidic conditions. (**d**) Cα RMSD traces from three independent 1-μs MD simulations of apo and ligand-bound CK-BB at pH 7.3 and pH 5.5, showing overall structural stability with greater conformational variability in the ligand-bound acidic state. (**e**) Per-residue root-mean-square fluctuation (RMSF) profiles for the same simulations. Shaded regions mark peptides with significant pH-dependent increases in deuterium uptake (red) and additional flexible segments (gray). Functional histidines and the Ser199 phosphorylation site are indicated by dashed lines. Bottom panels show RMSF values mapped onto CK-BB structures, with ribbon thickness proportional to local flexibility and HDX-sensitive regions highlighted in dark red. Together, the simulations support the HDX-MS data by showing that acid- and ligand-sensitive exchange regions either coincide with locally flexible segments or lie adjacent to dynamic elements that may modulate solvent exposure.

**Figure 9.**
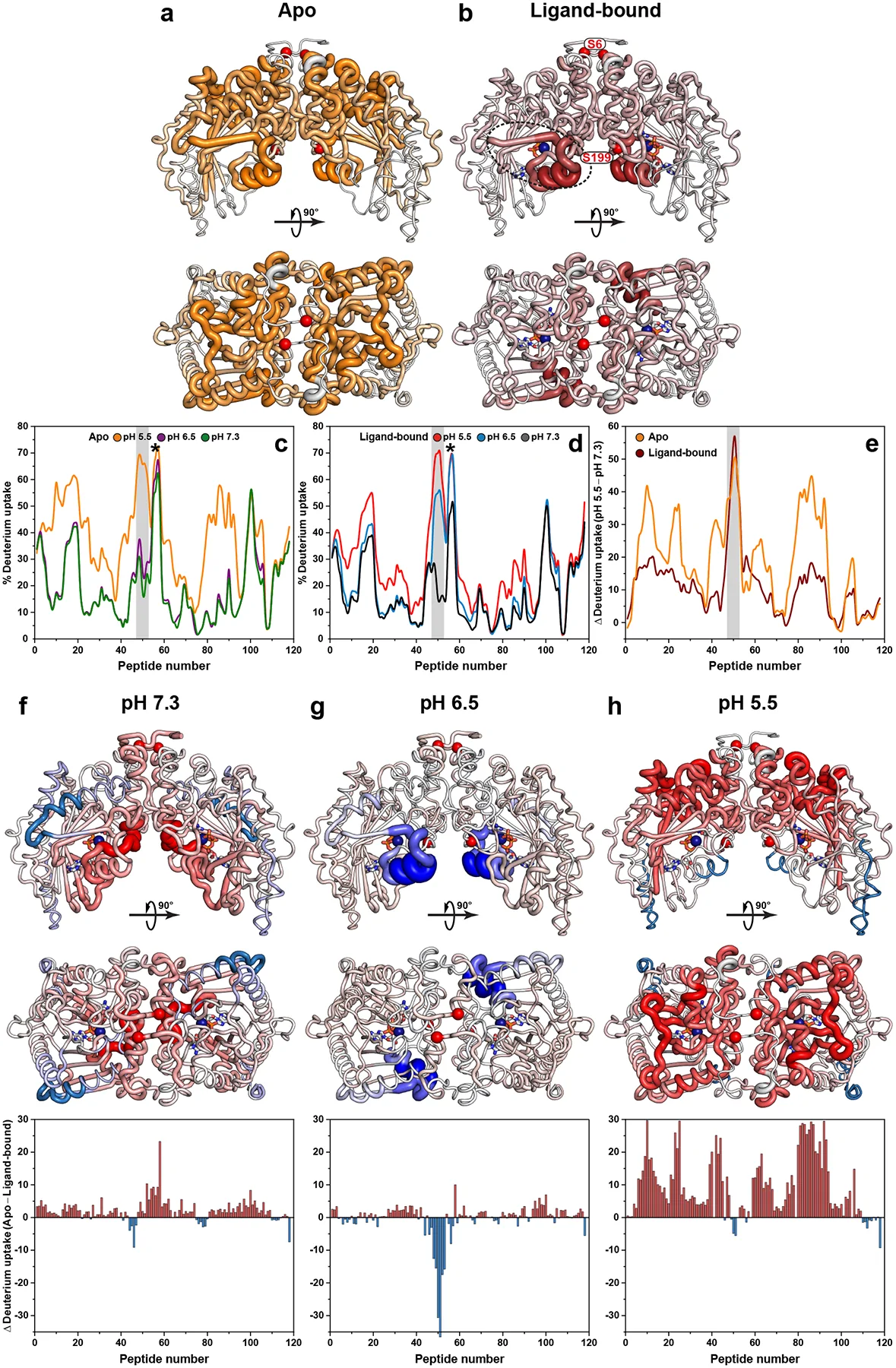
Ligand binding buffers global acid-induced CK-BB dynamics while sensitizing the His191/Ser199 region. (**a**,**b**) Differences in deuterium uptake at 60 s between pH 5.5 and pH 7.3 for apo (**a**) and ligand-bound (**b**) CK-BB, mapped onto the CK-BB dimer with ribbon thickness proportional to increased uptake under acidic conditions. In apo CK-BB, acidic pH broadly increases deuterium uptake across the NTDs, convex surface, and the His191/Ser199-containing concave region, whereas ligand binding substantially attenuates this global acid-induced deprotection while preserving a localized response near His191/Ser199. (**c**,**d**) Peptide-level uptake profiles across pH for apo (**c**) and ligand-bound (**d**) CK-BB. The shaded region marks peptides surrounding the short helix adjacent to the C-terminal beta-sheet core and preceding the Ser199 phosphorylation site (circled in **b**). In apo CK-BB, exchange at pH 6.5 remains closer to pH 7.3, whereas pH 5.5 produces a threshold-like increase in uptake. Ligand binding sensitizes this region to pH, producing graded increases in exchange across all three pH conditions. (**e**) Direct comparison of acid-induced uptake changes in apo and ligand-bound CK-BB highlights the global protective effect of ligand binding and the retained pH sensitivity of the His191/Ser199 region. (**f–h**) Differences in deuterium uptake at 60 s between apo and ligand-bound CK-BB at pH 7.3, 6.5 and 5.5, mapped onto the CK-BB dimer and shown as peptide-level bar plots. Positive values indicate greater uptake in apo CK-BB, consistent with ligand-dependent protection; negative values indicate greater uptake in the ligand-bound state. At pH 5.5, ligand binding strongly protects otherwise dynamic convex and NTD regions.

**Figure 10.**
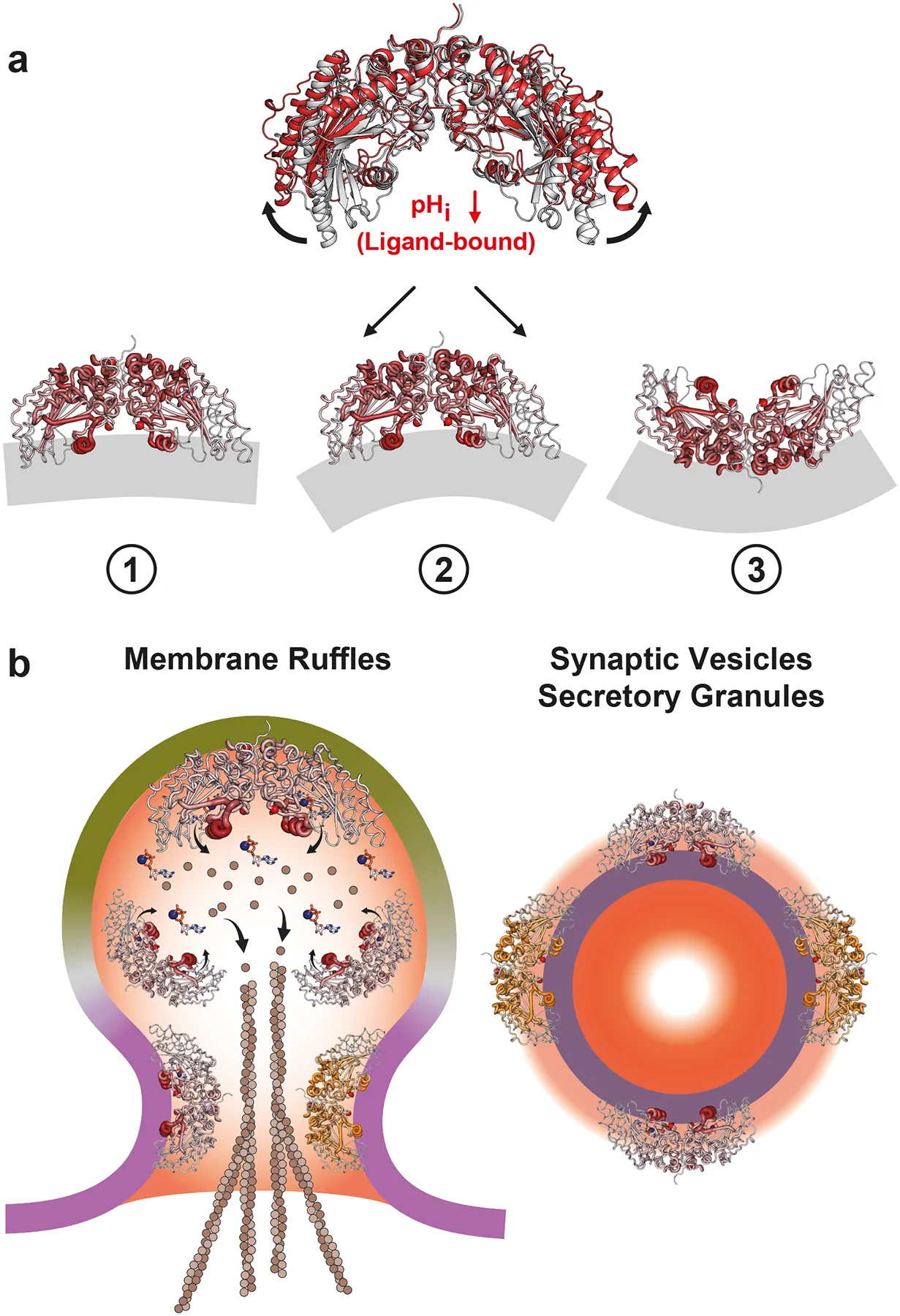
Model for acid- and ligand-regulated CK-BB coupling to vesicular and actin-remodeling membranes. Integrated model illustrating how acidic pH and substrate binding remodel CK-BB and promote its association with curved vesicular and actin-remodeling membranes. (**a**) DEER, HDX-MS and MD analyses support a model in which acidic pH, particularly in the ligand-bound state, enhances CK-BB dimer opening and increases dynamics across the convex surface and the His191/Ser199-containing concave region. Progressive dimer opening may facilitate CK-BB membrane association and adaptation to membrane curvature, providing a structural basis for recruitment to vesicular membranes, membrane ruffles and other curved cortical compartments. (**b**) Under acidic conditions, CK-BB redistributes from a largely cytosolic pool to punctate vesicular structures and membrane ruffles, where locally recruited CK-BB may regenerate ATP to support actin remodeling, membrane protrusion and vesicle-associated dynamics. Together, this model links acid- and ligand-dependent CK-BB conformational remodeling to localized ATP buffering at curved, actin-rich membrane domains.

## Data Availability

The generated data, including data from DEER, HDX-MS and native mass spectrometry experiments, are available in the manuscript or Supplementary Materials. Source data are provided with this paper. Additional datasets, including raw DEER data, MD input files, initial and final coordinates, analysis scripts, analysis data, DEER-guided AlphaFold models and refined models, have been deposited in the Zenodo repository maintained by CERN: https://doi.org/10.5281/zenodo.21539483. Other data supporting this study are available from the corresponding author upon request.
